# The Emerging Role of Zfp217 in Adipogenesis

**DOI:** 10.3390/ijms18071367

**Published:** 2017-06-27

**Authors:** Hong Xiang, Zhu-Xia Zhong, Yong-Dong Peng, Si-Wen Jiang

**Affiliations:** 1 Key Laboratory of Swine Genetics and Breeding of Agricultural Ministry, College of Animal Science and Technology, Huazhong Agricultural University, Wuhan 430070, China; hongxiang686@gmail.com (H.X.); zzx199320@163.com (Z.-X.Z.); pyd123456@163.com (Y.-D.P.); 2 The Cooperative Innovation Center for Sustainable Pig Production, Wuhan 430070, China; 3 Hebei Key Laboratory of Veterinary Preventive Medicine, College of Animal Science and Technology, Hebei Normal University of Science and Technology, Qinhuangdao 066004, China

**Keywords:** *Zfp217*, C3H10T1/2, 3T3-L1, adipogenesis, high fat diet (HFD), miRNAs

## Abstract

Zinc finger protein 217 (Zfp217), a member of the krüppel-type zinc finger protein family, plays diverse roles in cell differentiation and development of mammals. Despite extensive research on the functions of *Zfp217* in cancer, pluripotency and reprogramming, its physiological roles in adipogenesis remain unknown. Our previous RNA sequencing data suggest the involvement of *Zfp217* in adipogenesis. In this study, the potential function of *Zfp217* in adipogenesis was investigated through bioinformatics analysis and a series of experiments. The expression of *Zfp217* was found to be gradually upregulated during the adipogenic differentiation in C3H10T1/2 cells, which was consistent with that of the adipogenic marker gene *Pparg2*. Furthermore, there was a positive, significant relationship between *Zfp217* expression and adipocyte differentiation. It was also observed that *Zfp217* could not only trigger proliferative defect in C3H10T1/2 cells, but also interact with *Ezh2* and suppress the downstream target genes of *Ezh2*. Besides, three microRNAs (miR-503-5p, miR-135a-5p and miR-19a-3p) which target *Zfp217* were found to suppress the process of adipogenesis. This is the first report showing that *Zfp217* has the capacity to regulate adipogenesis.

## 1. Introduction

Obesity, a major global public health problem, is a complex disease that involves interactions between environmental and genetic factors [1]. The number of obesity patients keeps increasing year by year [statistical data by World Health Organization (WHO) official website], and related research has also experienced an explosive growth since 2011 (Figure S1). Hence, it will be of great importance to further study the adipocytic gene and explore the mechanism of obesity for therapeutic strategies.

Excess body adiposity is largely attributable to adipocyte hypertrophy and hyperplasia [2]. The hyperplasia is the result of excessive stem cells or preadipocytes differentiating into mature adipocytes (a process called adipogenesis). Adipogenesis is a complicated process that usually consists of six stages: mesenchymal precursor, committed preadipocyte, growth-arrested preadipocyte, mitotic clonal expansion, terminal differentiation, and mature adipocyte [2]. The adipogenic event involves a cascade of transcription factors and signaling pathways, with peroxisome proliferator-activated receptor g (Pparg) and CCAAT/enhancer-binding proteins (Cebps) considered as the crucial determinants of adipogenic fate [3].

In the past decades, despite reports about many important signaling pathways and certain essential transcriptional factors involved in adipogenesis, many adipogenic factors still remain to be excavated. Recent studies have revealed that a small but increasing number of zinc finger proteins could act as key transcriptional regulators in adipogenesis [4]. The zinc finger protein family is extremely abundant in higher eukaryotes and can function as sequence-specific DNA-binding factors in a variety of life processes. Previous studies discovered that *Shn-2*, *ZNF395*, *Zfp423*, *Zfp467*, *Zfp36L1*, *BCL6* and *Zfp521* have a pivotal role in adipogenic commitment [4,5,6]. *ZNF638*, *SLUG*, *Egr2*, *FBI-1*, *MCP-1*, *Hzf*, *MORC2*, *A20*, *Repin1*, *Zfp407*, *BCL11B* and *YY1* are positive regulators of preadipocyte differentiation [4,7,8,9,10,11,12,13,14], while *GATA2*, *GATA3*, *Egr1*, *Snail* and *PLZF* are reported as negative regulators of the transition from preadipocyte to adipocyte [4,15]. In addition, the majority of the zinc-finger protein family members of Krüpple-like factors are reported to promote or suppress preadipocyte differentiation [4,16]. In our previous work, differentially expressed genes (DEGs) were identified by RNA sequencing during the in vitro adipocytic differentiation of porcine subcutaneous stromal vascular cells (ASVC) on Days 0, 2 and 4, and *Zfp217* was found significantly upregulated at Day 2 during the adipogenesis [17]. It suggested that *Zfp217* was probably implicated in regulating cell adipogenic event.

Previous reports have suggested that *Zfp217* is the locus-amplification of chromosome 20q13.2 and is involved in multiple malignant tumors [18,19,20,21,22,23]. Besides, *Zfp217* has a pivotal role in the regulation of epithelial-to-mesenchymal transition (EMT), reprogramming, immortalization as well [24,25,26]. With the accumulation of research findings, *Zfp217* has been indicated as a key biomarker and regulator of carcinogenesis associated with poor prognosis [22,27,28]. Coincidentally, a number of zinc finger protein genes have clearly bilateral function in cancer and adipogenesis, such as *ZNF395*, *Zfp423*, *Zfp521*, *SLUG*, *Egr2*, *FBI-1*, *MCP-1*, *YY1*, and so on. [4,6,29,30,31,32,33,34,35,36,37]. However, this remains unknown for cases related to adipogenesis function, or the mechanism of *Zfp217*. Therefore, to further explore the adipocytic function of *Zfp217*, we examined expression profiling through high throughput sequencing or arrays in the Gene Expression Omnibus (GEO) datasets database. Additionally, the results from the bioinformatics analysis were tested by a series of experiments. This work shows that *Zfp217* plays a positive role in adipogenesis.

## 2. Results

### 2.1. The Potential Adipogenesis Role of Zfp217 Based on Gene Expression Omnibus (GEO) Datasets

**Figure 1:**
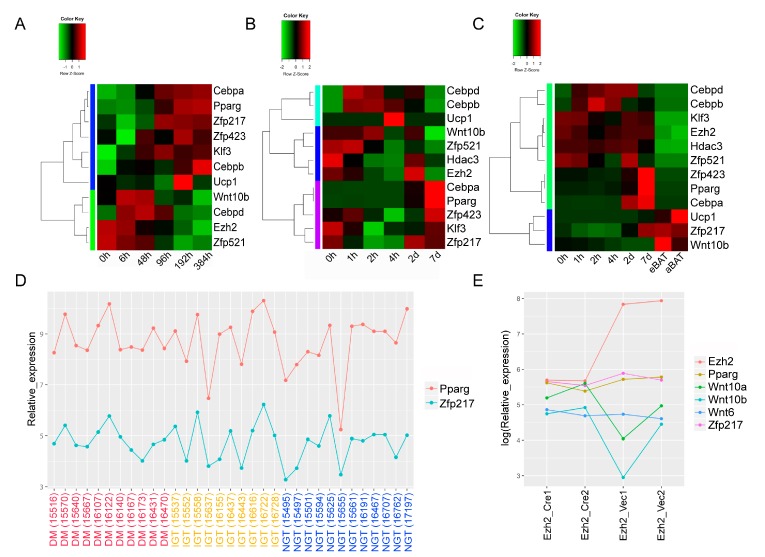
*Zfp217* is implicated in adipogenesis based on Gene Expression Omnibus (GEO) datasets. (**A**) The heatmap of expression profiling of *Zfp217* and marker genes in GSE76131. The resulting transcriptomic data-set includes six induction differentiation time points (0, 6, 48, 96, 192 and 384 h) in human simpson-golabi-behmel syndrome (SBGS) pre-adipocyte cells; (**B**,**C**) The heatmap of expression profiling of *Zfp217* and marker genes in GSE87113. Expression profiling by RNA-seq during adipogenesis (0, 1, 2, 4 h, 2 and 7 days) in 3T3-L1 (**B**) and brown preadipocytes (**C**) in culture as indicated in the *x* axis, eBAT: embryo_BAT; aBAT: adult_BAT; (**D**) The line chart of expression profiling of *Zfp217* and *Pparg* in diabetes mellitus (DM, the red group as indicated in the *x* axis), impaired glucose tolerance (IGT, the yellow group as indicated in the *x* axis) and normal glucose tolerance (NGT, the blue group as indicated in the *x* axis) adipose tissue samples. Data were obtained from GSE27951; and (**E**) The line chart of expression profiling of *Zfp217* and marker genes in GSE20054. Expression profiling by *Ezh2*-flox/flox preadipocytes infected with retroviruses expressing Cre or vector alone.

To investigate the potential roles of *Zfp217* in adipogenesis and obesity, we scanned differential expression genes associated with adipogenesis in GEO datasets, and found that the expression of *Zfp217* was highly correlated with marker genes during the adipogenesis process of model cells (Figure 1A–C). As shown in Figure 1A, the expression of *Zfp217* and *Pparg* was nearly consistent after adipogenic induction. Generally, obesity triggers multiple metabolic syndromes and is considered the most important predictor of diabetes mellitus. Obesity individuals show higher free fatty acid levels which stimulate insulin resistance, and then develop into diabetes mellitus [38]. Here, the expression of *Zfp217* fluctuated with that of *Pparg* in three kinds of individuals classified as having diabetes mellitus (DM), impaired glucose tolerance (IGT), and normal glucose tolerance (NGT) (Figure 1D), and this implied that the potential effect of *Zfp217* on obesity may be connected with *Pparg*. In addition, it found that *Zfp217* and *Pparg* showed a similar trend in *Ezh2* flox/flox preadipocytes when infected with retroviruses expressing Cre or vector alone (Figure 1E). The Wnt signaling genes are also changed with *Ezh2* as in previous research (Figure 1E).

### 2.2. The Association of Zfp217 Expression with Adipogenesis and Obesity

**Figure 2:**
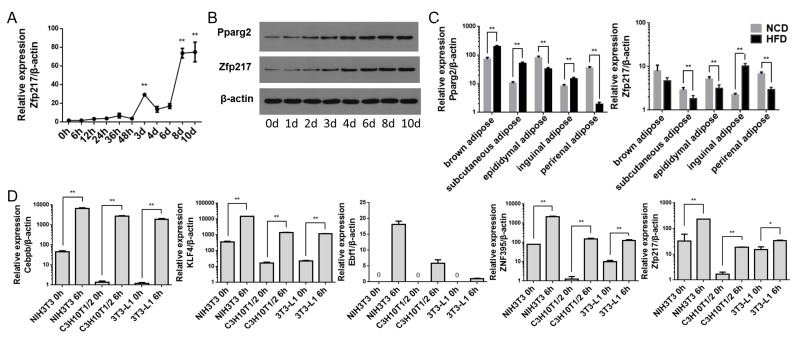
Relative expression of *Zfp217* in adipogenesis and different adipose tissues. (**A**) The expression profiles of *Zfp217* during C3H10T1/2 adipogenesis (0, 6, 12, 24, 36, 48 h, 3, 4, 6, 8 and 10 days) (*n* = 3); (**B**) The protein level of *Zfp217* and *Pparg2* during C3H10T1/2 adipogenesis (0, 1, 2, 3, 4, 6, 8 and 10 days); (**C**) Relative expression of *Pparg2* and *Zfp217* using five kinds of adipose tissues of HFD/NCD. The five adipose tissues as indicated in the *x* axis (*n* = 5); and (**D**) The mRNA level of *Zfp217* and pro-adipogenic marker genes (as indicated in the *y* axis) in NIH3T3, C3H10T1/2 and 3T3-L1 at non-differentiated and differentiated 6 h (*n* = 3). Data was standardized with *β-actin* and represented as means ± SEM. * *p* < 0.05, ** *p* < 0.01 versus control.

In order to explore the relationship between *Zfp217* expression and adipogenesis, we investigated the expression of *Zfp217* in non-differentiated C3H10T1/2 cells and during their differentiation into adipocytes. During adipogenesis, the mRNA level of *Zfp217* increased steadily, with an obvious peak at Day 3 (by 17-fold compared to 0 h; *p* < 0.01), followed by a decrease at Day 4, and then a significant increase during differentiation up to Day 8 and Day 10 (both by 43-fold compared to 0 h and *p* < 0.01) (Figure 2A). At the protein level, the expression of *Zfp217* was highly correlated with the extension of the induction time, and the protein expression pattern was similar to that of the adipogenic marker gene *Pparg2* (Figure 2B).

Furthermore, we separated five kinds of adipose tissues from high fat diet (HFD) and normal chow diet (NCD) fed mice based on *Zfp217*’s potential role in obesity, and tested the mRNA level of *Zfp217* and *Pparg2*. When compared with the NCD group, the mRNA level of *Zfp217* and *Pparg2* for the HFD group showed a similar trend in the visceral adipose tissues (which included epididymal, inguinal and perirenal adipose) (Figure 2C), but presented a reverse trend in the brown adipose and subcutaneous adipose (Figure 2C).

Meanwhile, in order to investigate the expression of *Zfp217* in cell models with different adipogenic ability, and the variation of *Zfp217* expression during the early stage of adipogenic induction, we examined the *Zfp217* expression levels in NIH3T3, C3H10T1/2 and 3T3-L1 at non-differentiation and 6 h after differentiation. Notably, the mRNA level of *Zfp217* was increased in all three cells at 6 h after differentiation (Figure 2D). We also investigated the mRNA level of early pro-adipogenic marker genes. Interestingly, both the expression patterns of *Zfp217* in the three cell models and at 6 h post-differentiation are similar to those of early pro-adipogenic marker genes (*Cebpb*, *KLF4*, *Ebf1* and *ZNF395*).

### 2.3. Zfp217 Regulates Adipogenesis of C3H10T1/2 and 3T3-L1

**Figure 3:**
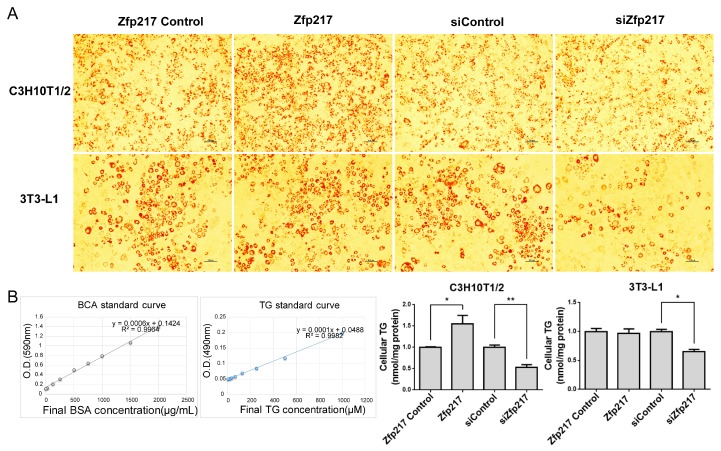
The effects of *Zfp217* “gain-of-function” and “loss-of-function” on lipid droplet formation and triglyceride content in C3H10T1/2 and 3T3-L1. (**A**) Oil red O staining of *Zfp217* “gain-of-function” and “loss-of-function” after 8 days of differentiation. Scale bar indicates 100 μm; and (**B**) Triglyceride GPO-POD assay kit (GPO: glycerol phosphate dehydrogenase; POD: peroxidase) and Bicinchoninic Acid (BCA) protein assay kit were used for analysis of the content of triglyceride. Total triglyceride was standardized with total protein and represented as means ± SEM (*n* = 4). * *p* < 0.05, ** *p* < 0.01 versus control.

In order to examine the effects of *Zfp217* on adipogenesis, siRNA-mediated loss-of-function and overexpression-mediated gain-of-function approaches were used to study the effect of *Zfp217* on the adipogenic differentiation of C3H10T1/2 and 3T3-L1. The mRNA level of *Zfp217* was significantly changed at Day 2 after transfection (Figure S2). Adipocyte differentiation was evaluated by Oil Red O staining and a triglyceride assay. As shown in Figure 3A, *Zfp217* knockdown resulted in an obvious decrease in the number of lipid droplets formed, both in C3H10T1/2 and 3T3-L1. Consistent with this observation, triglyceride contents were also significantly attenuated due to lack of *Zfp217* in C3H10T1/2 and 3T3-L1 (Figure 3B). Meanwhile, we also observed that *Zfp217* overexpression led to an induction of white adipocyte phenotype lipid droplet formation, a slight increase of the triglyceride content in 3T3-L1, and a remarkable increase in C3H10T1/2. These results imply that *Zfp217* is required for adipogenesis of the two types of adipogenic cell models.

### 2.4. Zfp217 Positively Regulates Adipogenesis by Suppressing Cell Proliferation and Interacting with Ezh2

**Figure 4:**
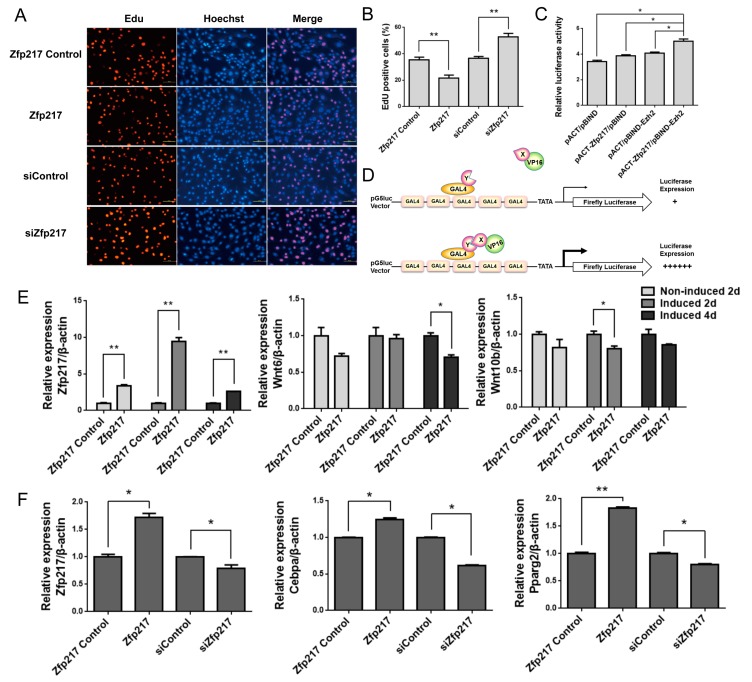
*Zfp217* suppresses cell proliferation and interacts with *Ezh2*, which represses Wnt signaling genes, to facilitate adipogenesis. (**A**) DNA synthesis of C3H10T1/2 cells was measured by 5-ethynyl-2′-deoxyuridine (EdU) incorporation assay after the indicated transfection. The EdU staining (red dots) represents the population of newborn cells. Hoechst 33342 staining (blue dots) was used to label cell nuclei. Scale bar indicates 100 μm; (**B**) EdU incorporation quantitative analysis. Five microscopic fields were randomly selected. EdU positive cells (%) were quantified by Image J, which was calculated by the formula: EdU positive cell (100%) = red dots/(red dots + blue dots) × 100%. All data were obtained from three reproducible experiments; (**C**) In the mammalian two-hybrid system, the *Zfp217*-*Ezh2* interaction is confirmed by significantly higher luciferase activity in cells transfected with pACT-*Zfp217* and pBIND-*Ezh2*, compared to cells transfected with one expression vector and one empty vector or two empty vectors. Results were displayed as firefly luciferase activity normalized to renilla luciferase activity and represented as means ± SEM (*n* = 4); (**D**) Schematic representation of the Mammalian Two-Hybrid System. The pG5luc Vector contains five GAL4 binding sites upstream of a minimal TATA box, which in turn is upstream of the firefly luciferase gene. The interaction between the two test proteins, expressed as GAL4-X and VP16-Y fusion constructs. An interaction between proteins X and Y brings the VP16 and GAL4 domains into close proximity, and results in an increase in luciferase expression over the negative controls; (**E**) The mRNA levels of Wnt6 and Wnt10b at non-induction, two days and four days post-adipogenic induction after treatment with pcDNA3.1-*Zfp217* or empty pcDNA3.1 vector (*n* = 3); and (**F**) The mRNA levels of *Cebpa* and *Pparg2* at four days post-adipogenic induction after treatment with pcDNA3.1-*Zfp217* or empty pcDNA3.1 vector/*Zfp217* siRNA or siRNA control (*n* = 3). Reverse transcriptase-quantitative polymerase chain reaction (qRT-PCR) data were standardized with *β-actin* and represented as means ± SEM. * *p* < 0.05, ** *p* < 0.01 versus control.

Given that *Zfp217* regulates adipogenesis with its loss-of-function and gain-of-function in C3H10T1/2, we further investigated the potential mechanism of *Zfp217* in C3H10T1/2. 5-Ethynyl-2′-deoxyuridine (EdU) assays were performed on cells transiently transfected separately with *Zfp217* siRNA or pcDNA3.1-*Zfp217* and its control. Our data indicated that *Zfp217* overexpression blocked DNA synthesis in C3H10T1/2, whereas *Zfp217* knockdown significantly increased EdU incorporation (*p* < 0.05) (Figure 4A,B). It was also observed that *Zfp217* could increase the amount of C3H10T1/2 cells in G0/G1-phase (Figure S3). That is, *Zfp217* arrested C3H10T1/2 cells at the G0/G1 phase, and *Zfp217* overexpression blocked DNA synthesis, which may be mediated by changes in the cell cycle.

According to previous reports and our bioinformatics analysis, we speculated that *Zfp217* may participate in adipogenesis by combining with *Ezh2*, which suppresses Wnts. In order to test this hypothesis, the open reading frame (ORF) of full-length mouse Zfp217 was cloned into the pACT expression vectors to generate VP16-Zfp217 chimeric proteins. Meanwhile, the ORF of full-length mouse *Ezh2* was cloned into the pBIND expression vectors to generate GAL4-Ezh2 chimeric proteins. Next, the interaction of *Zfp217* and *Ezh2* was tested. A robust interaction requires that the mammalian two-hybrid assay signal in pACT-*Zfp217*/pBIND-*Ezh2* should be significantly above background signals derived from both vectors individually (Figure 4D). We did find that the group of pACT-*Zfp217*/pBIND-*Ezh2* had a significant interaction signal comparable to control group (Figure 4C). Therefore, we can conclude that *Zfp217* also interacts with *Ezh2* in the mouse background.

Additionally, in order to confirm whether *Zfp217* represses Wnt signaling genes to facilitate adipogenesis, we checked the expression changes of Wnts (*Wnt6* and *Wnt10b*) and two major adipogenic marker genes (*Cebpa* and *Pparg2*) after treatment with *Zfp217*. It was observed that *Zfp217* overexpression could notably suppress the mRNA level of *Wnt6* at Day 4 post-adipogenic induction, and that of *Wnt10b* at Day 2 (Figure 4E), but the mRNA level of the two Wnt signaling genes was slightly decreased in the other time points. Furthermore, at Day 4 of post-adipogenic induction, the mRNA levels of *Cebpa* and *Pparg2* were significantly changed, with variation in *Zfp217* expression (Figure 4F). Hence, it can be concluded that *Zfp217* could positively regulate adipogenesis by suppressing cell proliferation and interacting with *Ezh2*, which represses Wnt signaling genes.

### 2.5. Direct Targeting of Zfp217 by miR-503-5p, miR-135a-5p and miR-19a-3p Impairs Adipocyte Differentiation

**Figure 5:**
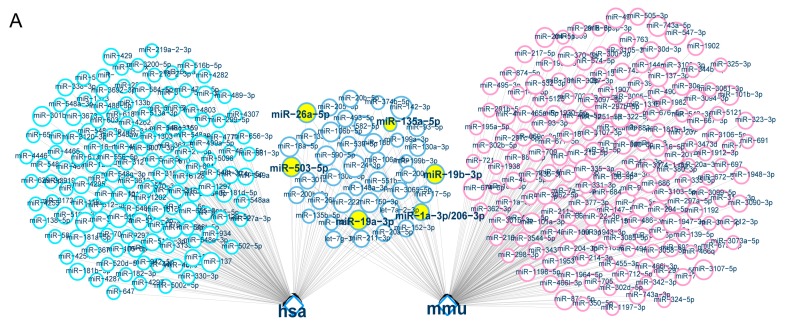

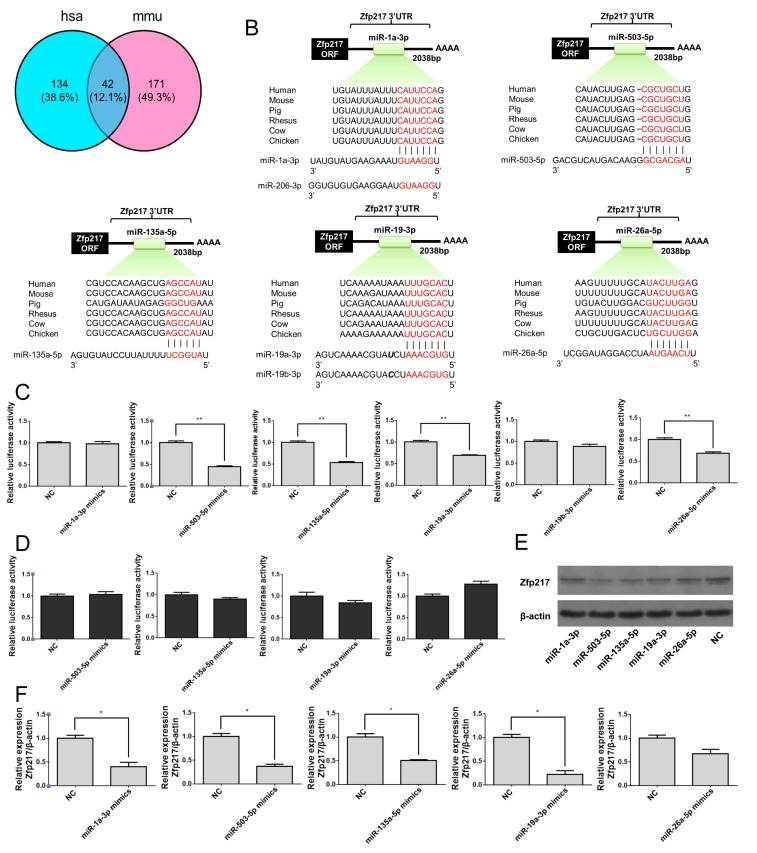
The miRNAs of targeting Zfp217. (**A**) The prediction of miRNAs which targeting *Zfp217*. The brilliant blue ring represents the predicted miRNAs in human (*Homo sapiens*, hsa). The pink ring represents the predicted miRNAs in mouse (*Mus musculus*, mmu). The gray blue ring represents the predicted miRNAs in both human and mouse. Venn diagram was output to statistical result; (**B**) Conservation of the miRNA binding site in the *Zfp217*-3′ unstranslated (UTR) region. The miRNA seed match region is highlighted in red; (**C**,**D**) Two pmirGLO vector constructs, containing either the *Zfp217*-3′ UTR-wt or the *Zfp217*-3′ UTR-mut with corresponding miRNA seed region, were transfected into BHK-21 cells either alone or in combination with negative control or each miRNA mimics. Renilla luciferase activity was used to normalize firefly luciferase activity. Data represents means ± SEM (*n* = 4); and (**E**,**F**) The expression levels of *Zfp217* was measured by Western blot and qRT-PCR for different treated groups as indicated. The qRT-PCR data represents means ± SEM (*n* = 3). * *p* < 0.05, ** *p* < 0.01 versus control.

Next, we predicted the miRNAs which targeting *Zfp217*, and conducted conservation analysis for the binding site of “seed region” by bioinformatics [39]. Then, we found that 176 miRNAs targeted *Zfp217* 3′ untranslated region (UTR) by binding the “seed region” in human, and 213 in mouse (Figure 5A). Forty-two miRNAs which were common between the two species, were further screened by Gene Ontology (GO) enrichment analysis to obtain adipogenic-miRNAs. Finally, we obtained five miRNAs (miR-1a-3p, miR-503-5p, miR-135a-5p, miR-19a/b-3p and miR-26a-5p). The binding sites of these mature miRNAs are highly conserved in poultry and mammals, including chicken, pig, human, rhesus monkey, mouse, and cow (Figure 5B).

To prove whether *Zfp217* was the direct target gene of those predicted miRNAs, 3′ UTR sequences containing the predicted “seed region” target site of miRNA and its mut-sequences were cloned into the pmirGLO vector. Using a dual-luciferase reporter system, the result showed significantly reduced luciferase activity in those co-transfected with the miRNAs (miR-503-5p, miR-135a-5p, miR-19a-3p and miR-26a-5p) mimics and pmirGLO-*Zfp217*-3′ UTR-wt (*p* < 0.01), but the group consisting of miR-1a-3p and miR-19b-3p did not display obvious changes (Figure 5C). Subsequently, we co-transfected with the pmirGLO-*Zfp217*-3′ UTR-mut and miRNAs mimics which showed changed activity. Unlike wild-type luciferase reporter, all four changed miRNAs expression could not inhibit the activity of the mut-reporter (Figure 5D). These results clearly indicate that the four miRNAs (miR-503-5p, miR-135a-5p, miR-19a-3p and miR-26a-5p) directly recognized and bound the 3′ UTRs of *Zfp217*.

To verify whether those miRNAs affected the *Zfp217* expression level, we transiently transfected C3H10T1/2 cells with miRNAs mimics and investigated *Zfp217* mRNA level at 24 h after transfection and protein level at 48 h after transfection. The results of reverse transcriptase-quantitative polymerase chain reaction (qRT-PCR) showed that over-expression of miRNAs (miR-1a-3p, miR-503-5p, miR-135a-5p and miR-19a-3p) dramatically suppressed the mRNA levels of *Zfp217* (*p* < 0.01) (Figure 5F), which was similar to western blot results (Figure 5E). Although miR-1a-3p could restrain *Zfp217* expression level, it could not bind the 3′ UTRs of *Zfp217* by “seed region”. On the contrary, miR-26a-5p could bind the 3′ UTRs of *Zfp217*, but it did not affect the *Zfp217* expression level.

**Figure 6:**
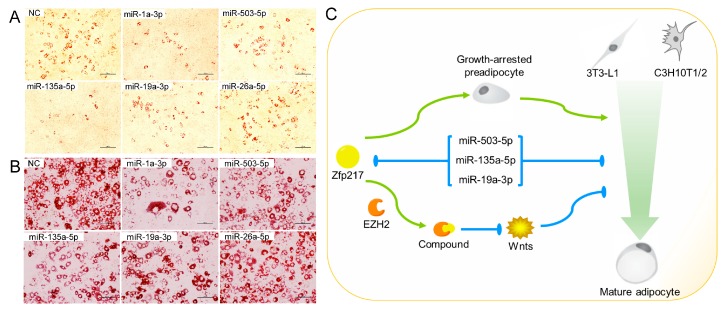
C3H10T1/2 (**A**) and 3T3-L1 (**B**) cells were stained with Oil Red O for different treated groups as indicated. One out of three independent but comparable experiments is shown. Scale bar indicates 100 μm; and (**C**) Schematic diagrams illustrating the mechanism of inducing adipogenesis by *Zfp217*. *Zfp217* positively regulates adipogenesis by suppressing cell proliferation and interacting with Ezh2 which represses Wnt signaling genes. *Zfp217* is directly targeted by miR-503-5p, miR-135a-5p and miR-19a-3p which impair adipocyte differentiation.

We also transiently transfected 3T3-L1 cells and C3H10T1/2 cells with the predicted miRNA mimics or negative control before adipogenic-induction to elucidate whether those miRNAs indeed regulate adipocyte differentiation. Compared with control group, miRNA (miR-1a-3p, miR-503-5p, miR-135a-5p and miR-19a-3p) mimic transfection yielded decreased lipid droplets accumulation in both two cells by Oil Red O staining (Figure 6A,B).

Together, the results showed that *Zfp217* is directly targeted by miR-503-5p, miR-135a-5p and miR-19a-3p which impair adipocyte differentiation. This is a further evidence of *Zfp217* has the effect on adipogenesis.

## 3. Discussion

Despite its participation in a variety of important life processes due to its special structure, the role of the zinc finger protein family in adipogenesis is poorly understood. In our previous study, we found that a differentially expressed zinc finger protein gene *Zfp217* was significantly upregulated at Day 2 during adipogenesis [17]. However, most of the previous studies are mainly focused on histone modification, pluripotency and oncogenesis [25,26,27,40,41], and its role in adipogenesis has not been reported. In this study, to test whether *Zfp217* plays a role in adipogenesis, we retrieved GEO datasets and performed a bioinformatics analysis of the downloaded datasets. We found that the expression of *Zfp217* is not only correlated with adipogenic marker genes, but also fluctuated with that of *Pparg* in three diabetes mellitus-related clinical statuses. Although the expression of *Pparg* is not obvious regularity in three clinical-status as the complex factors in vivo, the role of *Pparg* on obesity and diabetes mellitus is well known [42,43]. Therefore, we suspect that *Zfp217* may have a potential effect on obesity and its role may be under the control of *Pparg*. Furthermore, bioinformatics analysis and literature review suggest that *Zfp217* may affect adipogenesis by combining with *Ezh2*. Hence, a series of experiments were performed to test this hypothesis.

Previous studies have reported that *Pparg* acts as a transcriptional activator and has a central role in adipocyte differentiation, adipocyte metabolism and obesity [44]. *Pparg* is essential for both white and brown adipocytes differentiation under different conditions [42,45]. *Ucp1* is a marker gene of brown adipocyte [46]. The ectopic expression of *Ucp1* can increase the population of brown adipocytes [47], and *Ucp*1 can be also present in white adipose tissue [48]. Interestingly, in two types of white adipocytes (human SBGS pre-adipocyte and 3T3-L1 cells), *Zfp217* and *Pparg* are classified together. In brown preadipocytes, *Zfp217* and *Ucp1* classified more closely. This may imply that the relationship between *Zfp217* and *Ucp1* is closer than *Zfp217* and *Pparg* in brown preadipocytes. It suggests that *Zfp217* may have different regulatory mechanisms in different type of adipocytes. The specific mechanism of *Zfp217* in brown preadipocytes needs more detailed experiments to be verified. In this study, multiple mouse adipose tissues and cell lines were first used to analyze the expression patterns of *Zfp217* in the adipogenic process or the obesity model. With the extension of adipogenic induction time, *Zfp217* expression showed a steady increase, which was very similar to that of key adipogenic marker gene *Pparg2*. However, for the five kinds of different adipose tissues from HFD/NCD mice, the expression levels of *Zfp217* and *Pparg2* in brown adipose tissues were not consistent as those in visceral adipose tissues or white adipocytes, and this may have been due to many influencing factors in vivo, or other unknown regulatory mechanisms. Visceral adipocytes are crucial contributors to the burden of obesity and are more harmful to human health [49]. The mRNA level of *Zfp217* and *Pparg2* only had a similar pattern in cell adipogenesis and visceral fat, it may suggest that the role of *Zfp217* on white adipogenesis and obesity is closely related to *Pparg*. What’s more, *Pparg* has two transcription factor binding sites at the 5′ UTR of *Zfp217* sequence predicted using JASPAR datasets (Table S4), it implies that *Zfp217* may be the downstream gene of *Pparg*. The expression of *Pparg2* was not always increased in HFD group, suggesting that there was a protective mechanism against diet-induced weight gain. The protective mechanism may be induced by inflammation factors, DNA methylation or neuromodulation (as epididymal and perirenal tissues are richly endowed with nodose ganglions) [50,51,52]. *Zfp217* structure and properties were in accordance with early key pro-adipogenic genes. For the three cell models with different adipogenic ability before or after induction, the expression pattern of *Zfp217* was similar to that of early key pro-adipogenic genes. Meanwhile, the protein structure of *KLF4* and *ZNF395* also had zinc finger protein motifs. Furthermore, all four early key pro-adipogenic genes could be linked to cancer [29,53,54,55]. Therefore, the results of expression pattern analysis further supported the results of bioinformatics analysis.

In addition, “gain-of-function” and “loss-of-function” experiments further confirmed that *Zfp217* promoted adipogenesis in both C3H10T1/2 cells and 3T3-L1 cells. Consistent with this finding, *Zfp217* significantly positively regulated pro-adipogenic core transcription factors *Cebpa* and *Pparg2*. It seems that the increase of the *Zfp217* expression obviously decreased EdU incorporation, and vice versa. Since the cell cycle distribution is changed by *Zfp217* overexpression, this phenomenon is a result of the inhibition of mitotic clonal expansion. Namely, *Zfp217* could arrest C3H10T1/2 cells at G0/G1 phase and blocked DNA synthesis, and it turns out that *Zfp217* overexpression decreased EdU incorporation. This suggests that *Zfp217* regulates adipogenesis, probably by suppressing cell proliferation. In order to understand the potential molecular mechanism of *Zfp217* in adipogenesis, we surveyed the reports concerning *Zfp217* or adipogenesis in the past decades, and found that *Zfp217* was bound to *Ezh2* and participated in histone modification in MCF7, which is a breast cancer cell line [40]. Besides, *Ezh2* represses Wnt signaling genes to facilitate adipogenesis as well [56]. Concordant with our previous speculation, *Zfp217* was found to interact with *Ezh2* according to a mammalian two-hybrid assay. Furthermore, *Zfp217* overexpression was found to restrain the mRNA level of *Wnt6* and *Wnt10b*, the downstream target genes of *Ezh2* during the adipogenic differentiation process [56], both of which act as a negative molecular switch in governing adipogenesis [57]. Combining GEO analysis, we speculate that *Zfp217* could cooperate with *Ezh2* in facilitating adipogenesis.

We also found that three miRNAs (miR-503-5p, miR-135a-5p and miR-19a-3p) directly suppressed the expression levels of *Zfp217* by bonding its 3′ UTRs. Consistent with our findings, previous studies have also found that those miRNAs are related to adipogenesis. miR-503-5p inhibits adipogenesis by targeting *bmpr1a* in C3H10T1/2 cells [58], miR-135a-5p suppresses adipogenes by activating the canonical Wnt/β-catenin signaling in 3T3-L1 cells [59], and miR-19a-3p acts as a serum miRNAs biomarker for pancreatic cancer-associated new-onset diabetes mellitus [60] and it is also involved in C3H10T1/2 chondrogenic differentiation [61]. Based on our results and related reports, these three miRNAs impair adipocyte differentiation, and *Zfp217* is directly targeted by them; this in turn confirmed that *Zfp217* has an effect on adipogenesis.

Although our research remains to be advanced and improved upon, and although the role of *Zfp217* on brown adipogenesis is still indistinct, we have verified that *Zfp217* has a positive effect on white adipogenesis, through bioinformatics, literature mining and a series experiments. To the best of our knowledge, this is the first report showing that *Zfp217* promotes white adipogenesis in both C3H10T1/2 cells and 3T3-L1 cells, and that *Zfp217* may cooperate with *Ezh2* in facilitating its adipogenesis or triggering proliferative defects in C3H10T1/2 cells. Also three miRNAs which impair adipocyte differentiation are proposed to directly target *Zfp217* in the mouse background (Figure 6C).

## 4. Materials and Methods

### 4.1. Analysis of Gene Expression Omnibus Series (GSE) Data

All relevant GSE data were retrieved from GEO datasets (Table S1). The probe ID was converted into an Entrez Gene ID according to the Gene Expression Omnibus Platform (GPL) file. Adipogenesis-unrelated GSE samples were excluded. Differentially expressed genes were identified using the R package limma, with an Adj.P.Val < 0.05, and |log_2_ Fold Change| > 1 selected as the threshold for screening DEGs. The ggplot2 package was used for the visualization of the results.

### 4.2. Animals

Healthy male Kun Ming (KM) mice were housed in specific pathogen-free facilities on a 12/12 h light/dark cycle. Animals (four weeks of age) were randomized into chow diet (*n* = 6, researcher diets D12450B 10 Kcal %) and high-fat diet (*n* = 6, researcher diets D12492 60 Kcal %) groups. Animals were fed for ten weeks. Age-matched male littermates were used for all experiments. Five kinds of adipose tissues used in this study were carefully dissected from five depots [interscapular, epididymal, inguinal, perirenal white adipose tissue (WAT), and interscapular brown adipose tissue (BAT)], and immediately frozen in liquid nitrogen and stored at −80 °C for gene expression analysis. All experiments were performed in accordance with relevant guidelines and regulations. All experimental protocols were approved by the Ethics Committee of Huazhong Agricultural University with the permit number No. 30700571 for this study.

### 4.3. Cell Culture and Adipocyte Differentiation

3T3-L1 cells and C3H10T1/2 cells were purchased from the Type Culture Collection of the Chinese Academy of Sciences (Shanghai, China), and cultured in Dulbecco’s modified Eagle’s medium (DMEM, Gibco) with 10% fetal bovine serum (FBS, Gibco, Gaithersburg, MD, USA). The cells were induced to differentiation by addition of MDII cocktail (0.5 mM 3-isobutyl-1-methylxanthine, 1 μM dexamethasone, 5 μg/mL insulin and 100 μM indomethacin) in 10% FBS medium at 2 d after reaching confluence. Two days after induction, the cells were refed with 10% FBS medium with 5 μg/mL insulin for two days, then maintained in 10% FBS medium until cells were fully differentiated. NIH3T3 cells and BHK-21 cells were also cultivated in 10% FBS medium.

### 4.4. Oil Red O Staining

The fully differentiated cells were rinsed twice with PBS, and then fixed with 4% formalin for 30 min at room temperature. Then the cells were rinsed with PBS and stained with filtered Oil Red O (Sigma, Saint Louis, MO, USA) for 30 min, followed by washing twice in water. Stained cells were visualized by light microscopy (Nikon, Tokyo, Japan).

### 4.5. Triglyceride Assay

The fully differentiated cells were rinsed twice with PBS, collected in saline solution, and then sonicated for homogenization. The concentrations of triglyceride (TG) in the lysates of cells were measured with the commercial kits (Applygen, Beijing, China). The content of triglycerides was normalized to the content of protein (μmol/mg protein) using the bicinchoninic acid (BCA) assay kit (Thermo Scientific, Waltham, MA, USA).

### 4.6. Quantitative Real-Time RT-PCR (qRT-PCR) Analysis

Total RNA was obtained by using TRIzol (Invitrogen, Carlsbad, CA, USA). The cDNA was synthesized using RevertAid™ First Strand cDNA Synthesis Kit (Thermo Scientific) according to the manufacturer’s protocol. The qRT-PCR was performed in triplicate using IQ SYBR green Supermix (Bio-Rad, Hercules, CA, USA) on CFX384 (Bio-Rad). Relative quantification was calculated by the 2^−ΔΔ*C*t^ method and normalized by *β-actin*. Primers are listed in Table S2.

### 4.7. Western Blotting

Proteins were detected using antibodies against Zfp217 (sc-67223, Santa Cruz, Dallas, TX, USA), Pparg2 (sc-22020, Santa Cruz), and β-actin (sc-47778, Santa Cruz). Total cell lysate was extracted using RIPA lysis buffer on ice. Equal amounts of proteins were separated by SDS-PAGE electrophoresis. The protein bands were transferred to a polyvinylidene difluoride (PVDF) membrane (Millipore, Boston, MA, USA). The PVDF membrane was blocked with 5% skimmed milk and incubated with primary antibodies. The results were visualized using horseradish peroxidase-conjugated secondary antibodies (Santa Cruz) and enhanced chemiluminescence.

### 4.8. EdU Cell Proliferation Assay

The cell proliferation assay was carried out using EdU according to the manufacturer’s manual (RiboBio, Guangzhou, China). Briefly, cells (1 × 10^4^ cells/cm^2^) were cultured in 96-well plates and added final-concentration 50 μM EdU of each well for 4 h at 37 °C. Then, the cells were fixed using 4% paraformaldehyde for 30 min at room temperature and permeabilized with 0.5% Triton X-100 for 10 min. Next, the cells were washed with PBS and incubated with EdU staining solution for 30 min. Finally, the cells were re-stained with Hoechst 33,342 (200 μL per well) for 30 min. The cells were imaged under a fluorescent microscope (Nikon).

### 4.9. Mammalian Two-Hybrid Assay

Protein–protein interactions were assayed using the CheckMate™ Mammalian Two-Hybrid System (Promega, Madison, WI, USA). Briefly, BHK-21 cells were plated at a density of 1 × 10^5^ cells/cm^2^ in 48-well plates, cultured for 24 h and then co-transfected with the mixture of pACT- and pBIND-derived constructs or control vector, and a pG5luc reporter vector. The cells were passively lysed at 48 h post transfection. Firefly and renilla luciferase activities were measured using the Dual-Luciferase^®^ Reporter Assay System (Promega) and an EnSpire Multimode Plate Reader with two automated injectors (PerkinElmer, Norwalk, CT, USA).

### 4.10. The Prediction and Screening of miRNAs

The miRNAs which targeting *Zfp217* were predicted by miRNAWalk2.0 at the specific cut-off criteria (SUM ≥ 6, Targetscan = True) [39]. It extracted data from the overlap-miRNAs both in human and mouse. Conservative-overlap miRNAs were screened out from the overlap-miRNAs using targetscan. Base on the targeting principle of miRNAs, the GO annotation analysis of the conservative-overlap miRNAs was performed using their reverse prediction program targetgenes and performed by DAVID v6.8 [62].

### 4.11. Dual-Luciferase Reporter Assay

The 3′ UTR fragments of *Zfp217* were amplified from the mouse genome. The corresponding mutant fragments were amplified by overlap extension PCR. The primers and mutant sites were as shown in Table S3 and Figure S4. The PCR fragments were cloned into pmirGLO vectors (Promega). All vectors were confirmed by sequencing. BHK-21 cells were plated at a density of 1 × 10^5^ cells/cm^2^ in 48-well plates and then co-transfected with of the 3′ UTR-pmirGLO vectors (wt or mut) and the miRNAs mimics (The sequences were listed in Table S2) or negative control (GenePharma, Shanghai, China) using Lipofectamine 2000 (Invitrogen). The dual-luciferase activity was measured utilizing the Dual-Glo Luciferase Assay System (Promega). Firefly luciferase activity was normalized to the corresponding Renilla luciferase activity. Experiments were performed in quadruplicate wells of a 48-well plate and repeated at least three times.

### 4.12. Statistical Analysis

Data values were presented as mean ± SEM. Statistical analyses were performed using two-tailed Student’s *t*-tests. *p* < 0.05 was considered as statistically significant.

## 5. Conclusions

In this study, we reported a new adipogenic activator *Zfp217* and proposed a new mechanism of adipogenesis. The data presented in this study not only provide an insight into the adipogenesis regulatory networks, but also suggest that *Zfp217* may be an important therapeutic target in obesity.

## Supplementary Materials

Supplementary materials can be found at www.mdpi.com/1422-0067/18/7/1367/s1.

## Abbreviations

*Zfp217*	Zinc Finger Protein 217
*Pparg*	Peroxisome Proliferator-Activated Receptor g
Cebps	CCAAT/Enhancer-Binding Proteins
DEGs	Differentially Expressed Genes
ASVC	Adipocytic Differentiation of Porcine Subcutaneous Stromal Vascular Cells
GEO	Gene Expression Omnibus
GSE	Gene Expression Omnibus Series
GPL	Gene Expression Omnibus Platform
DM	Diabetes Mellitus
IGT	Impaired Glucose Tolerance
NGT	Normal Glucose Tolerance
HFD	High Fat Diet Fed Mice
NCD	Normal Chow Diet Fed Mice
ORF	Open Reading Frame
EdU	5-Ethynyl-2’-Deoxyuridine
TG	Triglycerides
BCA	Bicinchoninic Acid
wt	Wild Type
mut	Mutant Type
hsa	*Homo sapiens* (human)
mmu	*Mus musculus* (Mouse)
WAT	White Adipose Tissue
BAT	Brown Adipose Tissue
PVDF	Polyvinylidene Difluoride
EMT	Epithelial-to-Mesenchymal Transition
SBGS	Simpson-Golabi-Behmel Syndrome
GO	Gene Ontology
KM	Kun Ming
