# Supplementary material for: The Emerging Role of Zfp217 in Adipogenesis
Source: Int J Mol Sci. 2017 Jun 27;18(7):1367. doi: 10.3390/ijms18071367 (PMC5535860; doi:10.3390/ijms18071367)
Supplement: Supplementary file 1 [file ijms-18-01367-s001.pdf]

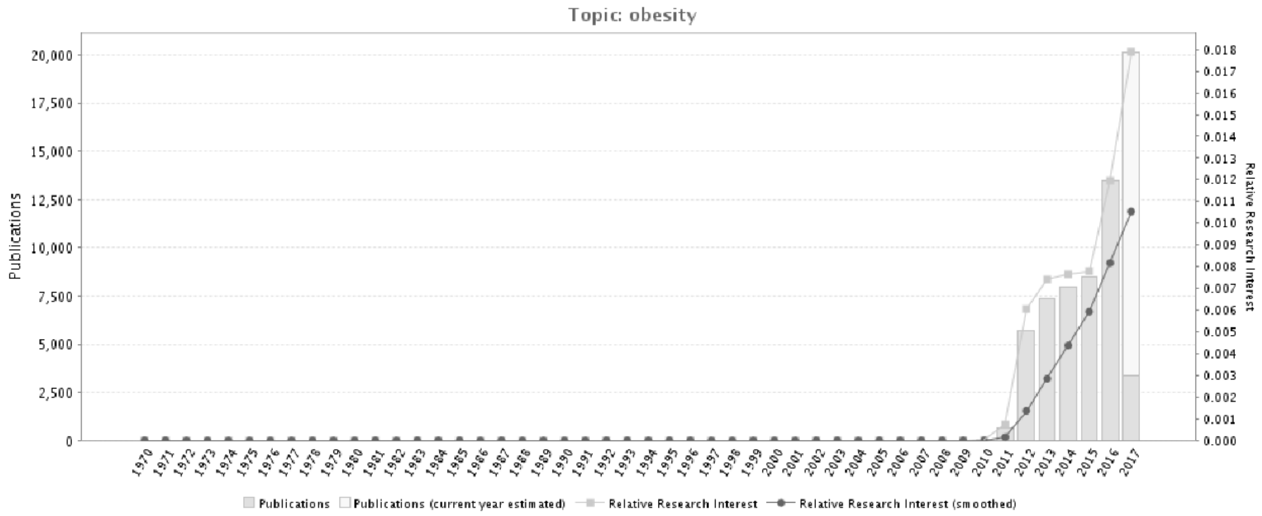

Figure S1. Data concerning obesity research since 2011. Data statistics were based on online publications and output by GoPubmed (http://www.gopubmed.com/web/gopubmed/).

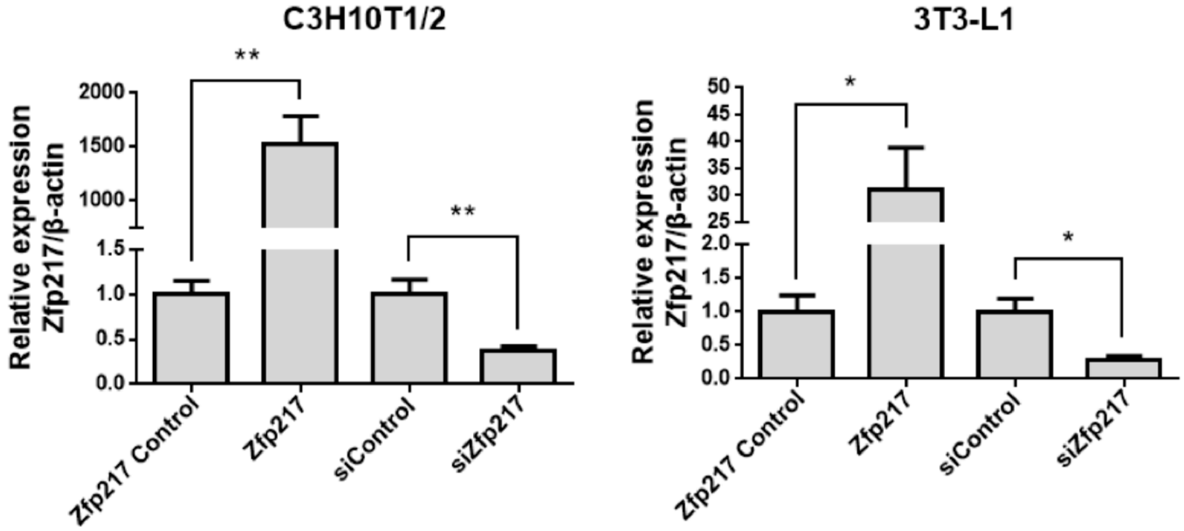

Figure S2. The mRNA levels of Zfp217 was measured by reverse transcriptase-quantitative polymerase chain reaction (qRT-PCR) at 48 h after transfection for different treated groups as indicated in C3H10T1/2 and 3T3-L1. Data represents means ± SEM. * $p < 0.05$, ** $p < 0.01$ versus control.

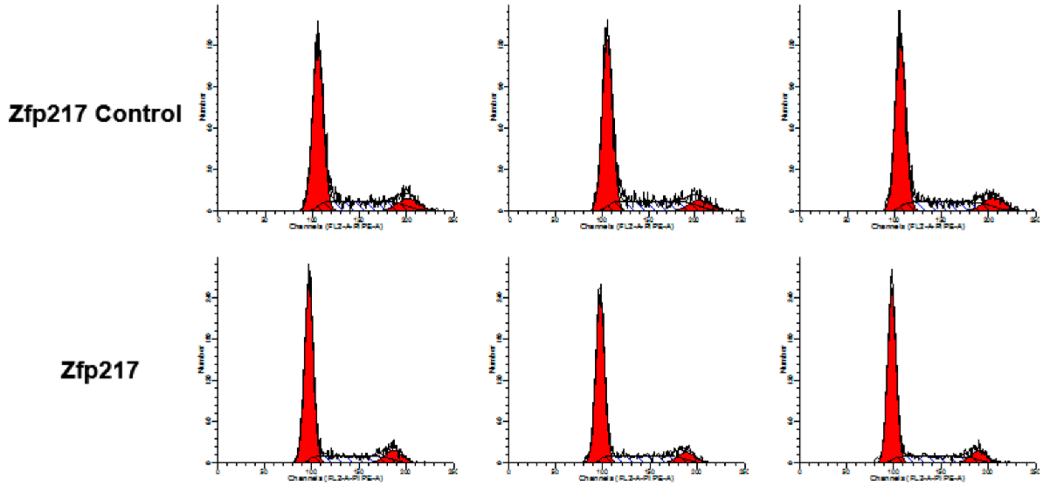

	G0/G1	S	G2/M
Zfp217 Control	64.50%	25.73%	9.77%
	64.60%	26.95%	8.45%
	64.61%	24.81%	10.58%
Zfp217	69.51%	20.77%	9.72%
	68.62%	22.68%	8.70%
	69.32%	21.41%	9.27%

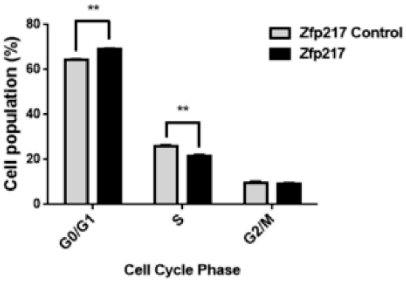

Figure S3. The effect of Zfp217 overexpression on the cell cycle phase of C3H10T1/2 cells. After 48 h transfection, C3H10T1/2 cells were detected by flow cytometry and the cell cycle phase was analyzed. Data represents means ± SEM. * $p < 0.05$, ** $p < 0.01$ versus control.

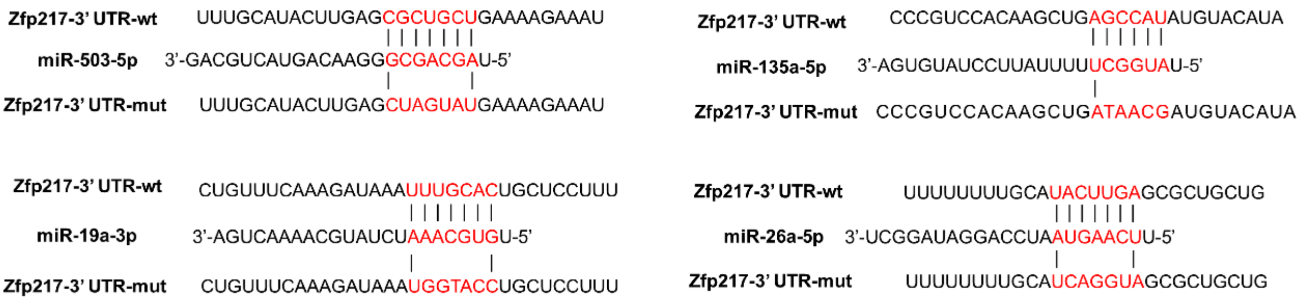

Figure S4. The microRNA (miRNA) binding region sequences of Zfp217-3′ untranslated region (UTR) of wild type (wt) and mutant type (mut). The miRNA seed match region is highlighted in red.

Table S1. The information of Gene Expression Omnibus (GEO) datasets used in this study.

GEO Accession	Platform	Overall design	Samples	Organism	References
GSE87113	GPL17021	Expression profiling by RNA-seq during adipogenesis (0 h, 1 h, 2 h, 4 h, 2 d, 7 d) in 3T3-L1 and brown preadipocytes in culture. Expression profiling by RNA-seq in brown adipose tissues of adult mice or embryos	GSM2322558 (RNA_3T3-L1_0 h), GSM2322559 (RNA_3T3-L1_1 h), GSM2322560 (RNA_3T3-L1_2 h), GSM2322561 (RNA_3T3-L1_4 h), GSM2322562 (RNA_3T3-L1_2 d), GSM2322563 (RNA_3T3-L1_7 d), GSM2322564 (RNA_brown preadipocytes_0 h), GSM2322565 (RNA_brown preadipocytes_1 h), GSM2322566 (RNA_brown preadipocytes_2 h), GSM2322567 (RNA_brown preadipocytes_4 h), GSM2322568 (RNA_brown preadipocytes_2 d), GSM2322569 (RNA_brown preadipocytes_7 d), GSM2322570 (RNA_adult_BAT), GSM2322571 (RNA_embryo_BAT)	*Mus musculus*	[1]
GSE76131	GPL10558	The adipogenesis process of human SBGS pre-adipocyte cells in vitro was monitored by obtaining gene expression profiles at beginning of experiment and at different time points after induction of differentiation. The resulting transcriptomic data set includes six time points (0, 6, 48, 96, 192, 384 hours), with three to nine replicates per time point, for a total of 26 profiles	GSM1974849 (0 h sample, replicate 1), GSM1974850 (0 h sample, replicate 2), GSM1974851 (0 h sample, replicate 3), GSM1974852 (6 h sample, replicate 1), GSM1974853 (6 h sample, replicate 2), GSM1974854 (6 h sample, replicate 3), GSM1974855 (6 h sample, replicate 4), GSM1974856 (6 h sample, replicate 5), GSM1974857 (6 h sample, replicate 6), GSM1974858 (6 h sample, replicate 7), GSM1974859 (6 h sample, replicate 8), GSM1974860 (6 h sample, replicate 9), GSM1974861 (48 h sample, replicate 1), GSM1974862 (48 h sample, replicate 2), GSM1974863 (48 h sample, replicate 3), GSM1974864 (96 h sample, replicate 1), GSM1974865 (96 h sample, replicate 2), GSM1974866 (96 h sample, replicate 3), GSM1974867 (96 h sample, replicate 4), GSM1974868 (192 h sample, replicate 1), GSM1974869 (192 h sample, replicate 2), GSM1974870 (192 h sample, replicate 3), GSM1974871 (384 h sample, replicate 1), GSM1974872 (384 h sample, replicate 2), GSM1974873 (384 h sample, replicate 3), GSM1974874 (384 h sample, replicate 4)	*Homo sapiens*	[2]

GSE27951	GPL570	Expression profiling by Affymetrix microarray for Homo sapiens subcutaneous adipose tissue.<br>Adipose tissue biopsies were obtained from the subcutaneous abdominal region (from the area below the umbilicus in a 4-cm range on either side) using the percutaneous needle biopsy technique with suction. Biopsies were quickly dissected free from visible blood and connective tissue and frozen in liquid nitrogen. To avoid interference of circadian rhythms and post-prandial responses, biopsies were obtained between 8 am and 10 am following an overnight fast	GSM691122 (adipose_tissue (15495), clinical status: NGT, BMI: 38.1, age: 22), GSM691123 (adipose_tissue (15497), clinical status: NGT, BMI: 35.3, age: 48), GSM691124 (adipose_tissue (15501), clinical status: NGT, BMI: 32.9, age: 39), GSM691125 (adipose_tissue (15516), clinical status: DM, BMI: 42.2, age: 56), GSM691126(adipose_tissue (15537), clinical status: IGT, BMI: 39.7, age: 41), GSM691127 (adipose_tissue (15552), clinical status: IGT, BMI: 36.7, age: 47), GSM691128 (adipose_tissue (15558), clinical status: IGT, BMI: 41.5, age: 58), GSM691129 (adipose_tissue (15570), clinical status: DM, BMI: 50.2, age: 49), GSM691130 (adipose_tissue (15594), clinical status: NGT, BMI: 37.4, age: 46), GSM691131 (adipose_tissue (15625), clinical status: NGT, BMI: 36.7, age: 45), GSM691132 (adipose_tissue (15637), clinical status: IGT, BMI: 39.5, age: 57), GSM691133 (adipose_tissue (15640), clinical status: DM, BMI: 27.2, age: 50), GSM691134 (adipose_tissue (15655), clinical status: NGT, BMI: 33.2, age: 60), GSM691135 (adipose_tissue (15661), clinical status: DM, BMI: 25.1, age: 60), GSM691136 (adipose_tissue (15667), clinical status: DM, BMI: 23.2, age: 55), GSM691137 (adipose_tissue (16107), clinical status: DM, BMI: 27.8, age: 59), GSM691138 (adipose_tissue (16122), clinical status: DM, BMI: 31.8, age: 51), GSM691139 (adipose_tissue (16140), clinical status: IGT, BMI: 26.9, age: 77), GSM691140 (adipose_tissue (16155), clinical status: DM, BMI: 27.4, age: 67), GSM691141 (adipose_tissue (16167), clinical status: DM, BMI: 28.8, age: 64), GSM691142 (adipose_tissue (16173), clinical status: DM, BMI: 32.2, age: 58), GSM691143 (adipose_tissue (16191), clinical status: NGT, BMI: 23.6, age: 37), GSM691144 (adipose_tissue (16431), clinical status: DM, BMI: 39.1, age: 44), GSM691145 (adipose_tissue (16437), clinical status: IGT, BMI: 34.2, age: 44), GSM691146 (adipose_tissue (16443), clinical status: IGT, BMI: 33.1, age: 59), GSM691147 (adipose_tissue (16467), clinical status: NGT, BMI: 26.4, age: 27),	*Homo sapiens*	[3]

			GSM691148 (adipose_tissue (16470), clinical status: DM, BMI: 25.1, age: 62), GSM691149 (adipose_tissue (16616), clinical status: IGT, BMI: 27.3, age: 59), GSM691150 (adipose_tissue (16707), clinical status: NGT, BMI: 26.2, age: 57), GSM691151 (adipose_tissue (16722), clinical status: IGT, BMI: 16.7, age: 59), GSM691152 (adipose_tissue (16728), clinical status: IGT, BMI: 23.0, age: 69), GSM691153 (adipose_tissue (16762), clinical status: NGT, BMI: 23.1, age: 42), GSM691154 (adipose_tissue (17197), clinical status: NGT, BMI: 23.2, age: 64)		
GSE20054	GPL1261	Affymetrix microarray analysis in Ezh2flox/flox preadipocytes infected with retroviruses expressing Cre or vector alone	GSM501363 (Ezh2-Cre1), GSM501364 (Ezh2-Cre2), GSM501365 (Ezh2-Vec1), GSM501366 (Ezh2-Vec2)	*Mus musculus*	[4]

BAT: Brown adipose tissue. DM: Diabetes mellitus, IGT: Impaired glucose tolerance, NGT: Normal glucose tolerance. The diagnostic criteria of DM, IGT and NGT are according to Alberti et al. [5].

Table S2. The sequences of reverse transcriptase-quantitative polymerase chain reaction (qRT-PCR) primers, microRNAs (miRNAs) and small interfering RNAs (siRNAs) used in this study.

**qRT-PCR primers**	
**Gene name**	**Primer sequence (5'-3')**
mmu-Zfp217	F: CTCTCTAGGCTCCCAGATGGA<br>R: AGAACATGCAATCCAGGGGC
mmu-PPARγ2	F: TTCCATGCTGTCATGGGTGA<br>R: AAAGTTGGTGGGCCAAAACG
mmu-Cebpα	F: CTCCCAGAGGACCAATGAAA<br>R: AAGTCTTAGCCGGAGGAAGC
mmu-Cebpβ	F: CCAAGAAGACGGTGGACAAGC<br>R: CTTGAACAAGTTCCGCAGGGT
mmu-Wnt6	F: GCGGAGACGATGTGGACTTC<br>R: ATGCACGGATATCTCCACGG
mmu-Wnt10b	F: GCTGACTGACTCGCCCACCG<br>R: AAGCACACGGTGTTGGCCGT
mmu-KLF4	F: TACCCCTACACTGAGTCCCG<br>R: GGAAAGGAGGGTAGTTGGGC
mmu-Ebf1	F: ATCCAACGGAGTGGAAGCAG<br>R: GCCAGGACGAAGTGGAAGAA
mmu-ZNF395	F: CGGTGGAGATGGACGAGATG<br>R: GCTCTCCTTCCACAGGTCAC
mmu-β-actin	F: TGTCCACCTTCCAGCAGATGT<br>R: AGCTCAGTAACAGTCCGCCTAGA

**Zfp217 siRNA or miRNA**	
**name**	**sense sequence (5'-3')**
Zfp217-Mus-3563	GCUGCAGAAGAGAAGCUAU
miR-1a-3p	UGGAAUGUAAAGAAGUAUGUAU
miR-503-5p	UAGCAGCGGGAACAGUACUGCAG
miR-135a-5p	UAUGGCUUUUUAUUCCUAUGUGA
miR-19a-3p	UGUGCAAAUCUAUGCAAAACUGA
miR-19b-3p	UGUGCAAAUCCAUGCAAAACUGA
miR-26a-5p	UUCAAGUAAUCCAGGAUAGGCU

Table S3. The sequences of dual-luciferase assay primers used in this study.

name	Primer sequence (5'-3')
Zfp217-3' UTR (miR-1a-3p)	F: AGCTTT*GTTTAAAC*GGCGCGCCGGAGCCCTTGTGCTTCAGTATG
	R: CCG*CTCGAG*CGGAAAGCATGTCTACTGACAACT
Zfp217-3' UTR (miR-503-5p)	F: AGCTTT*GTTTAAAC*GGCGCGCCGGAAAGGTCCCTGGATGCAAGT
	R: CCG*CTCGAG*CGGCCAAGCACGCAATTCACACA
	Mutant-F: TGCATACTTGAGC**TAGTA**TGAAAAG
	Mutant-R: TGATTTCTTTTCA**TACTA**GCTCAAG
Zfp217-3' UTR (miR-135a-5p)	F: AGCTTT*GTTTAAAC*GGCGCGCCGGTGCTCCTTTTTCTTGCCCCA
	R: CCG*CTCGAG*CGGTATGTGACCCCCTCCGGATT
	Mutant-F: CCACAAGCTGA**TAACG**ATGTAC
	Mutant-R: CTTATGTACAT**CGTTA**TCAGCT
Zfp217-3' UTR (miR-19a/b-3p)	F: AGCTTT*GTTTAAAC*GGCGCGCCGGTTGAGTCTGGGTGAGTTGCT
	R: CCG*CTCGAG*CGGACATCTCAACCCAGGTCTCC
	Mutant-F: CAAAGATAAAT**GGTAC**CTGCTCC
	Mutant-R: AGGAGCAG**GTACC**ATTTATC
Zfp217-3' UTR (miR-26a-5p)	Wild type primers are as same as miR-503-5p
	Mutant-F: TTTGCAT**CAGGT**AGCGCTGC
	Mutant-R: GCAGCGCT**ACCTG**ATGCAAA

Italics represent enzyme loci and bold represents mutation sites.

Table S4. Putative sites predicted in the 5' UTR Zfp217 sequence.

Model name	Score	Relative score	Start	End	Strand	predicted site sequence
Pparg::Rxra	9.369	0.82329080866099	460	474	1	TTAAGGGAAATGTCA
Pparg::Rxra	8.552	0.809986605370543	706	720	1	CCTGGTCAGAGGGTT
Pparg::Rxra	10.370	0.839591307429334	942	956	-1	GTGAGGCAGGGATCA

References

1. Park, Y.K.; Wang, L.; Giampietro, A.; Lai, B.; Lee, J.E.; Ge, K. Distinct roles of transcription factors KLF4, Krox20, and peroxisome proliferator-activated receptor γ in adipogenesis. *Mol. Cell. Biol.* **2017**, *37*, doi:10.1128/MCB.00554-16.
2. Nassiri, I.; Lombardo, R.; Lauria, M.; Morine, M.J.; Moyseos, P.; Varma, V.; Nolen, G.T.; Knox, B.; Sloper, D.; Kaput, J.; et al. Systems view of adipogenesis via novel omics-driven and tissue-specific activity scoring of network functional modules. *Sci. Rep.* **2016**, *6*, 28851.
3. Keller, P.; Gburcik, V.; Petrovic, N.; Gallagher, I.J.; Nedergaard, J.; Cannon, B.; Timmons, J.A. Gene-chip studies of adipogenesis-regulated microRNAs in mouse primary adipocytes and human obesity. *BMC Endocr. Disord.* **2011**, *11*, 7.
4. Wang, L.; Jin, Q.; Lee, J.E.; Su, I.H.; Ge, K. Histone H3K27 methyltransferase Ezh2 represses *Wnt* genes to facilitate adipogenesis. *Proc. Natl. Acad. Sci. USA* **2010**, *107*, 7317–7322.
5. Alberti, K.G.; Zimmet, P.Z. Definition, diagnosis and classification of diabetes mellitus and its complications. Part 1: Diagnosis and classification of diabetes mellitus provisional report of a who consultation. *Diabet. Med.* **1998**, *15*, 539–553.
